# Determination of five endosulfan pesticides in the fish pond water by dispersive liquid–liquid microextraction combined with GC–MS

**DOI:** 10.1080/20961790.2016.1278111

**Published:** 2017-01-30

**Authors:** Fangmin Xu, Lingyun Liu, Wanli Wei, Ruolun Xu

**Affiliations:** a Institute of Forensic Science, Public Security Bureau of Jiangyin, Jiangyin, China; b Institute of Forensic Science, Public Security Bureau of Wuxi, Wuxi, China

**Keywords:** Forensic science, forensic toxicology, gas chromatography–mass spectrometry, liquid–liquid microextraction, analytic sample preparation methods, endosulfan, pesticides, fish pond water

## Abstract

A simple and rapid dispersive liquid–liquid microextraction (DLLME) technique coupled with gas chromatography–ion trap mass spectrometry (GC–MS) was developed for the extraction and analysis of five endosulfan pesticides from the fish pond water. In this work, different parameters affecting the extraction process such as the type and volume of extraction solvent, type and volume of disperser solvent, and extraction time were studied and optimized. Under optimized conditions, the enrichment factor ranged from 189 to 269 and the relative recovery ranged from 88.5% to 94.9%. The linear range was 2.0–80.0 µg/L; the limits of detection and quantitation were in the range 0.04–1.06 µg/L and 0.12–3.53 µg/L, respectively. The relative standard deviations were in the range 0.94%–2.08% (*n* = 5). The obtained results show that DLLME combined with GC–MS is a fast and simple method for the determination of endosulfan pesticides in fish pond water.

## Introduction

Endosulfan, also called thiodan, is an organochlorine pesticide that is extensively used in agricultural production [1]. The two isomers, endo and exo, are known popularly as α and β [2]. Because of its toxicity to human health and the environment, some countries had banned its manufacture and use [3]. However, in a few other countries, such as China, it is still used. Therefore, some cases about endosulfan, for example poisoning, suicide, have occurred constantly, especially poisoning cases on the fish pond water. But the concentration of endosulfan pesticides in the water is very low in these cases. Conventional extraction methods such as liquid–liquid extraction (LLE) [4] and solid-phase extraction (SPE) [5] require large volumes of organic solvents and are time-consuming. To deal with these disadvantages, solid-phase microextraction (SPME) has been developed. SPME [6] uses no extraction solvent, but it is also expensive, its fibre is fragile and has limited lifetime and sample carry-over can be a problem. In recent years, a few new preconcentration technologies have been introduced, such as single-drop microextraction (SDME) [7], hollow-fibre-protected liquid-phase microextraction (HF-LPME) [8], supercritical fluid extraction (SFE) [9], and molecularly imprinted polymer-based solid-phase micro-extraction (MIP-SPME) [10]. All of these techniques have their own advantages; however, there can also be relatively expensive and long extraction times. It is important to develop a sensitive and simple preconcentration method for the determination of endosulfan pesticides in the fish pond water.

In 2006, Sana Berijani and coworkers have developed a novel microextraction technique as a preconcentration method, which they have named as dispersive liquid–liquid microextraction (DLLME) [11]. The advantages of the DLLME are simplicity of operation, rapidity, low cost, high recovery and enrichment factors (EFs).

In this work, a DLLME sample preparation method was developed for the preconcentration of five endosulfan pesticides from fish pond water. Several parameters of the extraction process including the type and volume of extraction solvent, type and volume of disperser solvent, and extraction time were optimized, and the developed method was applied for real fish pond water analysis.

## Experimental

### Reagents and standards

Endosulfan-α (EA, purity, 99.4%), endosulfan-β (EB, purity, 99.4%), endosulfan ether (EE, purity, 99.0%), endosulfan sulfate (ES, purity, 99.0%), and endosulfan lactone (EL, purity, 99.0%) were purchased from J&K Chemical Ltd., Shanghai. Tetrachloroethylene (purity, 99%), chloroform (purity, 99%), carbon tetrachloride (purity, 99.5%), and chlorobenzene (purity, 99%) were obtained from J&K Chemical Ltd., Shanghai. These solvents were distillated at least four times and were used as extraction solvents. Acetone (purity, 99.5%), acetonitrile (purity, 99.9%), methanol (purity, 99.9%) and ethanol (purity, 99.9%) were obtained from Sigma, Shanghai, which were distillated at least four times and were used as disperser solvent.

### Instrumentation

Analysis of endosulfan pesticides was performed on a Varian GC CP-3800 Saturn 2200 GC-MS system. Ultrapure helium (99.99%) was passed through a water trap and oxygen trap before its use as the carrier gas. The GC CP-3800 was fitted with a VF-5ms column (30 m×0.25 mm i.d., 0.25 µm) obtained from Agilent Technologies. Helium was used as the carrier gas at a flow rate of 1.0 mL/min. The oven temperature program employed for separation of endosulfan herbicides was as follows: 120 °C for 1 min; 10 °C/min to 260 °C, held for 2 min; then 20 °C/min to 280 °C, held for 2 min, and the 1177 split/splitless injector was made in the splitless mode. The ion trap mass detector was used in the electron impact (EI, 70 eV) mode and full scanned over the range *m*/*z* 50–450 to confirm the retention time of the analytes. The ion trap and transfer line temperatures were 150 and 230 °C, respectively. A Sorvall TDL-80-2B (Shanghai Anting Scientific Instrument Factory, China) was used for centrifuging.

### Preparation of standard solutions

10.0 mg of each endosulfan pesticide was dissolved in 10 mL methanol to obtain a standard stock solution with a concentration of 1.0 mg/mL and stored at −18 °C. Each fresh 100 µg/mL standard solution containing an endosulfan pesticide was prepared in methanol every week and stored at −18 °C. The working solutions were prepared daily by using standard solutions with suitable dilutions. Water samples used for evaluation of the method were collected in glass bottle from the Yangtze River, and stored at 4 °C.

### DLLME procedure

**Figure 1.**
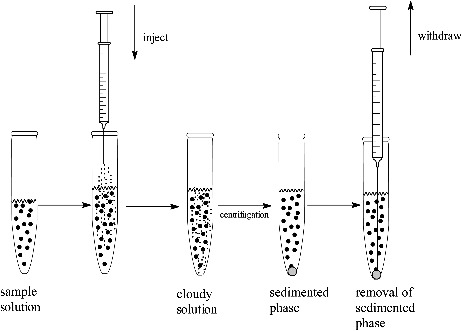
The procedure of dispersive liquid-liquid microextraction.

The experimental procedure for DLLME is illustrated in Figure 1. Five milliliter water was placed in a 10 mL glass test tube with conical bottom and spiked with each endosulfan pesticide at suitable concentration. The disperser solvent, containing extraction solvent, was rapidly injected into the sample solution with a syringe, and a cloudy solution (water, disperser solvent, and extraction solvent) was formed in the test tube; the cloudy state was stable for a long time. The mixture was let stand for 2 min, and then centrifuged for 5 min at 4 000 r/min, causing the dispersed droplets of the extraction phase to settle to the bottom of the conical test tube. The 2.0 µL of sediment extraction phase was collected using a 10-µL microsyringe and injected into the gas chromatography–ion trap mass spectrometry (GC-MS). The volume of the sediment phase was determined using a 100-µL microsyringe.

### Calculation of enrichment factor and extraction recovery

The EF was defined as the ratio between the analyte concentration in the sedimented phase (*C*_sed_) and the initial concentration of analyte (*C*_0_) within the sample:

$$EF=\frac{C_{\text{sed}}}{C_{0}}.$$

The concentration of analyte was obtained from calibration graph of direct injection of standard solution at the suitable range.

The extraction recovery (ER) was defined as the percentage of the total analyte amount (*n*_0_) which was extracted to the sedimented phase (*n*_sed_).

$$ER=\frac{n_{\text{sed}}}{n_{0}}\times 100=\frac{C_{\text{sed}}\times V_{\text{sed}}}{C_{0}\times V_{0}}\times 100=\text{EF}\times \frac{V_{\text{sed}}}{V_{0}}\times 100,$$

where *V*_sed_ and *V*_0_ are the volumes of sedimented phase and sample solution, respectively.

## Results and discussion

The extraction efficiency of DLLME procedure depends on some important experimental parameters which should be investigated. The effects of type and volume of extraction solvent, type and volume of disperser solvent, and extraction time were studied.

### Selection of extraction solvent and disperser solvent

**Figure 2.**
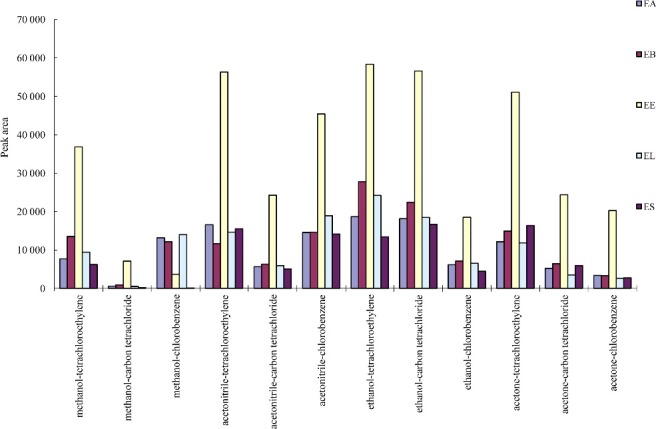
Efficiency of different extraction solvent and disperser solvent evaluated for extraction of endosulfan pesticides by DLLME. Extraction conditions: sample volume, 5.00 mL, extraction solvent volume, 20 μL, disperser solvent volume, 1.0 mL, room temperature; concentration of each endosulfan pesticides, 1.0 μg/mL.

The selection of an appropriate solvent is more important for the DLLME process. Extraction solvents are selected on the basis of higher density rather than water, extraction capability of interested compounds, and good gas chromatography behaviour. In this study, tetrachloroethylene, chloroform, carbon tetrachloride and chlorobenzene were compared in the extraction of endosulfan pesticides. Dispersive solvents should be miscible solvents with both aqueous samples and extraction solvents to help the analytes transfer from aqueous phase into organic phase. Acetone, acetonitrile, methanol and ethanol were studied as dispersive solvents. Thus, the series of solvents were compared for the extraction of the studied endosulfan pesticides, and were evaluated for extraction using the following model: 5.0 mL of sample spiked with each endosulfan pesticide at concentration of 1.0 μg/mL, 1.0 mL of dispersive solvent and 20.0 μL of extraction solvent were used. The extraction efficiency was evaluated by comparison of the peak area of each analyte. The peak area of each analyte is shown in Figure 2.

For chloroform, no cloudy solution was observed and no separated phases were obtained after centrifugation, and hence chloroform was rejected. The results revealed that the series of ethanol (dispersive solvent) and tetrachloroethylene (extraction solvent) has the highest extraction efficiency in comparison with the other series. Thereby, tetrachloroethylene and ethanol were selected as the extraction solvent and dispersive solvent, respectively.

### Optimization of extraction solvent volume

**Figure 3.**
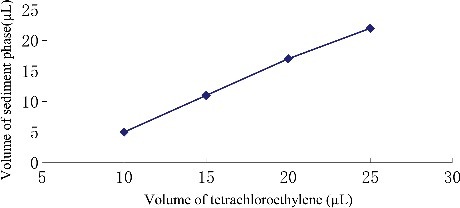
Effect of the volume of tetrachloroethylene on the volume of sediment phase in DLLME. Extraction conditions: sample volume, 5.00 mL, extraction solvent volume, 20 μL, disperser solvent volume, 1.0 mL, room temperature; concentration of each endosulfan pesticides, 1.0 μg/mL.

**Figure 4.**
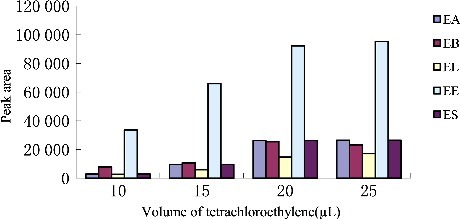
Efficiency of the volume of tetrachloroethylene evaluated for extraction of endosulfan pesticides by DLLME. Extraction conditions: sample volume, 5.00 mL, extraction solvent volume, 20 μL, disperser solvent volume, 1.0 mL, room temperature; concentration of each endosulfan pesticides, 1.0 μg/mL.

To study the effect of extraction solvent volume, solutions containing different volumes of tetrachloroethylene were subjected to exactly the same DLLME procedure. The experimental conditions were fixed and included the use of a constant volume of ethanol (1.0 mL) containing different volumes of tetrachloroethylene (5.0, 10.0, 15.0, 20.0 and 25.0 µL). Figures 3 and 4 show the curve of volume of sediment phase and the histogram of peak area versus volume of tetrachloroethylene, respectively. According to Figure 3, by increasing the volume of tetrachloroethylene from 5.0 to 25.0 µL, the volume of the sediment phase increases from 0 to 22.0 µL. For 5.0 µL, no separated phases were obtained after centrifugation, and it was rejected. Regarding Figure 4, by increasing the volume of tetrachloroethylene, the peak areas increased due to increase in the volume of organic phase collected after extraction which in turn leads to increase in analytes concentrations into the organic phase. When the volume of tetrachloroethylene was 20.0 µL, peak areas appeared to plateau, which indicates the quantitative extraction and high distribution coefficients of endosulfan pesticides in this condition. Thereby, the good sensitivity was achieved by using 20.0 µL of tetrachloroethylene.

### Optimization of disperser solvent volume

**Figure 5.**
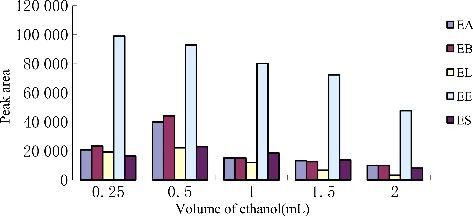
Efficiency of the volume of ethanol evaluated for extraction of endosulfan pesticides by DLLME. Extraction conditions: sample volume, 5.00 mL, extraction solvent volume, 20 μL, disperser solvent volume, 1.0 mL, room temperature; concentration of each endosulfan pesticides, 1.0 μg/mL.

In this study, changing volume of the disperser solvent might be effective on the extraction efficiency. Hence, to obtain the optimum volume of the disperser solvent, various volumes of ethanol (0.25, 0.5, 1.0, 1.5, 2.0 mL) containing 20.0 µL tetrachloroethylene were tested. The results are shown in Figure 5. According to the histogram, the extraction efficiency of mass analytes first increased, and then decreased by increasing the volume of ethanol. It seems, at a low volume of ethanol, cloudy state is not formed well, and thereby the ER decreases. At the high volume of ethanol, the solubility of endosulfan pesticides in water increases; therefore, the extraction efficiency decreases. A 0.5 mL of ethanol was chosen as optimum volume.

### Effect of extraction time

Time is the most important factor in the mass transfer of analytes from sample solution to the extraction solvent; therefore, this factor was evaluated in the paper. In DLLME, extraction time is defined as the time interval between injecting the mixture of disperser solvent containing extraction solvent and starting to centrifuge. In the constant experimental conditions, the effect of time was set at 0.5, 1, 2, 5, and 10 min, respectively.

**Figure 6.**
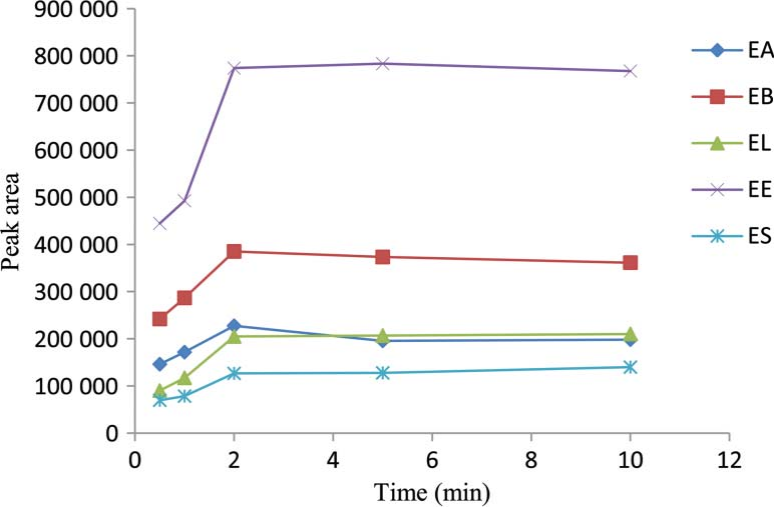
Efficiency of the extraction time evaluated for extraction of endosulfan pesticides by DLLME. Extraction conditions: sample volume, 5.00 mL, extraction solvent volume, 20 μL, disperser solvent volume, 1.0 mL, room temperature; concentration of each endosulfan pesticides, 1.0 μg/mL.

Figure 6 shows the peak areas of endosulfan pesticides versus extraction time. Because of the infinitely large surface area between extraction solvent and aqueous phase after the formation of cloudy solution, the mass transfer of analytes is so fast that the extraction equilibrium can be achieved in a short time. According to the curve, the peak area of endosulfan pesticides increases quickly, which reaches the maximum and then plateaued. Therefore, 2 min was chosen as optimum time.

### Quantitative analysis

Under optimized conditions, the proposed method was applied to the analysis of the endosulfan pesticides in the water; the retention time and ions selected for monitoring of analytes are shown in Table 1. Analytical characteristics of the method were evaluated in determination of endosulfan pesticides according to the DLLME process under the optimized conditions. Some analytical features such as EF, linear range, squared correlation coefficient, limit of detection, limit of quantification and repeatability were investigated. Table 2 summarizes the analytical characteristics of the optimized method. Linearity of calibration curve was observed at the range of 2.0–80.0 µg/L for most of the analytes. Coefficient of correlation (*r*) ranged from 0.994 6 to 0.996 8. The repeatability and recoveries were studied by extracting the samples containing each endosulfan pesticide at 20.0 µg/L. The EF of endosulfan pesticides was from 189 to 269 and the ER was from 68.0% to 96.8%, the relative recovery was from 88.5% to 95.1%. The relative standard deviations (RSDs) were calculated to be in the range of 0.94%–2.08% for five repeated experiments. The limits of quantification (LOQs), based on signal-to-noise ratio (S/N) of 10, ranged from 0.12 to 3.53 µg/L, and the limits of detection (LODs), based on S/N of 3, ranged from 0.04 to 1.06 µg/L, which is very low by using GC–MS.

**Table 1.** Retention time and ions selected for monitoring of analytes.

Analyte	Retention time (min)	Molecular mass	Monitoring ions (*m*/*z*)
EE	10.699	342.9	69^a^, 277, 241
EL	12.675	356.8	321^a^, 277, 239
EA	13.433	406.9	267^a^, 339, 241
EB	14.561	406.9	267^a^, 339, 195
ES	15.293	422.9	387^a^, 272, 229

a Most abundant ion.

**Table 2.** Quantitative features of the method for the endosulfan pesticides.

Analyte	Linear range (µg/L)	*r*	RSDs (*n* = 5) (%)	LOQs (µg/L)	LODs (µg/L)	EF	ER (%)	Relative recovery (%)
EA	2.0–80.0	0.995 9	0.94	0.19	0.06	243	87.5	90.4
EB	2.0–80.0	0.994 6	0.97	0.19	0.06	269	96.8	94.9
EL	2.0–80.0	0.996 0	1.60	0.21	0.06	213	76.7	89.8
EE	2.0–80.0	0.996 4	1.91	0.12	0.04	225	81.0	95.1
ES	4.0–80.0	0.996 8	2.08	3.53	1.06	189	68.0	88.5

### Real forensic sample analysis

In October 30 2014, a lot of fishes were found dead in a fish pond, which is located in a small village. It is estimated that the damage was over 70 000 RMB. The fish pond water was submitted for detection in our lab, and pretreatment by LLE, SPE and DLLME, respectively.

Table 3 shows that LLE and SPE required large volumes of organic solvents, were time-consuming, and had an unsatisfactory result. However, the optimized DLLME procedures only required 15 min, and provided a satisfactory result. The retention time confirmed the existence of EA and EB in the fish pond water, and the concentration were 7.8 and 3.2 µg/L, respectively.

**Table 3.** Comparison of the three pretreatment methods of fish pond water.

Method	Organic solvent	Volume (mL)	Pretreatment time (min)	Volume of the sample (mL)	Result
LLE	Dichloromethane	50	210	300	None
SPE	Methanol	10	240	50	EA, EB
DLLME	Ethanol	1	15	5	EA, EB

## Conclusion

In this study, a simple, rapid, and inexpensive microextraction technique has been coupled with a GC–MS method for the determination of five endosulfan pesticides in the fish pond water. The optimum conditions of extraction have been obtained. And the experimental results reveal that this method provides high extraction efficiency within a short time compared to other techniques, good selectivity and repeatability, low LODs and LOQs, and good linearity over the investigated concentration range. The method is also applied for the extraction of endosulfan from fish pond water, with good results. Comparison of this new method with other extraction methods such as LLE and SPE shows that DLLME is simple, rapid, of high efficiency, and inexpensive. Therefore, it has the potential to be a powerful tool for the analysis of endosulfan pesticides in forensic samples.
